# 共价有机骨架功能材料及其在糖肽选择性富集中的应用

**DOI:** 10.3724/SP.J.1123.2021.02001

**Published:** 2021-06-08

**Authors:** Qianying SHENG, Yang ZHOU, Zhiquan ZHAO, Yaohui WANG, Weicheng LI, Yanxiong KE, Minbo LAN, Guangyan QING, Xinmiao LIANG

**Affiliations:** 1.华东理工大学化学与分子工程学院, 上海 200237; 1. School of Chemistry and Molecular Engineering, East China University of Science and Technology, Shanghai 200237, China; 2.华东理工大学药学院, 上海 200237; 2. School of Pharmacy, East China University of Science and Technology, Shanghai, 200237, China; 3.中国科学院大连化学物理研究所, 辽宁 大连 116023; 3. Dalian Institute of Chemical Physics, Chinese Academy of Sciences, Dalian 116023, China

**Keywords:** 质谱, 共价有机骨架材料, 功能化修饰, 糖肽, 选择性富集, mass spectrometry (MS), covalent organic framework (COF) material, functional modification, glycopeptides, selective enrichment

## Abstract

蛋白质糖基化是生物体中最重要的翻译后修饰手段之一,糖蛋白/糖肽的有效分离和富集成为目前糖蛋白组学研究的首要问题。对于复杂的生物样本,糖蛋白的数量较少,酶解后大量高丰度非糖基化修饰肽的存在,使得低丰度糖肽的检测更加困难。因此,需要一些手段来有效地富集糖肽以提高其检测丰度,发展高选择性的糖肽富集材料及方法就成为在分子水平上有效地监测糖蛋白或糖肽的重要途径。相对于传统的糖肽富集材料,共价有机骨架材料具有比表面积大和可修饰位点丰富的优点,在糖肽富集领域具有很大的应用潜力。该文制备了一种新型的共价有机骨架材料(O-T-D-COFs),利用1,3,5-三(4-氨苯基)苯和2,5-二甲氧基苯-1,4-二甲醛作为反应单体通过共聚缩合反应生成的席夫碱构成了材料的框架,对合成后的中间体材料进行氧化处理,从而提高材料的富集性能。利用扫描电镜、透射电镜、红外光谱和固体核磁等表征技术对材料的结构进行了表征,并将其应用于糖肽的选择性富集。分别对富集过程的上样条件、淋洗条件、洗脱条件进行了优化,结合质谱检测技术,从人血清免疫球蛋白G酶解液中观察到32个明显的糖肽信号峰。通过模拟复杂样本体系验证材料富集选择性,在人血清免疫球蛋白G和牛血清白蛋白的酶解液混合物摩尔比达到1∶50时,该材料仍然保持了良好的选择性。此外,还考察了材料的检测限、富集容量、回收率等富集性能,及在实际样品中的应用潜力。以人血清免疫球蛋白G为评价对象,O-T-D-COFs具有较低的检测限(2.5 fmol/μL)、较高的富集容量(120 mg/g),及较好的富集回收率(103.5%±6.6%、101.5%±10.4%)。在血清样品中富集到来自53个*N*-糖蛋白中的86个*N*-糖肽序列,并鉴定到了94个*N*-糖基化位点。这些结果都表明,该材料在糖肽富集领域有较好的应用前景。

## Abstract (en)

Protein glycosylation is among the most important post-translational modifications in living organisms and the research in the field of protein glycosylation continues to garner attention. Currently, the efficient separation and enrichment of glycoproteins and glycopeptides is the primary challenge of glycoproteomics research. The number of glycoproteins is small in complex biological samples. Moreover, the presence of highly-abundant, non-glycosylated, and modified peptides makes the detection of low-abundance glycopeptides more difficult. Therefore, efficient glycopeptide enrichment methods are required to improve the detection of these compounds. The development of highly selective glycopeptide enrichment tools is important to efficiently capture glycoproteins or glycopeptides at the molecular level. Compared with traditional glycopeptide-enriched materials, covalent organic framework materials have the advantages of large specific surface area and rich modification sites, thereby exhibiting great application potential in the field of glycopeptide enrichment. In this study, a novel covalent organic framework material (O-T-D-COFs) was prepared and applied for selective glycopeptide enrichment. We applied the solvothermal method, using 2,5-dimethoxy benzene-1,4-2 formaldehyde and 1,3,5-Tris(4-aminophenyl) benzene, to synthesize imino-based COFs. The Schiff base generated via copolymerization condensation reaction constitutes the framework of the material. Next, the synthesized intermediate material was oxidized to improve the enrichment performance of the material. The functional, specific glycopeptide-binding groups were modified on the COF channels and the structure of the material was characterized using scanning and transmission electron microscope, as well as infrared spectrum and solid-state nuclear magnetic resonance. The enrichment conditions comprised the loading and elution steps, including the optimization of the elution conditions. We could observe the clear profile of 32 glycopeptides derived from human serum immunoglobulin G (IgG) tryptic digests with a significantly improved signal-to-noise (*S/N*) ratio. We applied a complex sample system to verify the enrichment selectivity of the material when the molar ratios of the IgG and bovine serum albumin (BSA) tryptic digest mixtures reached 1∶50. In addition, we investigated the enrichment performance of the detection limit, enrichment capacity, recovery rate of the material, and the application potential in glycopeptides enrichment using real samples. The material showed a good detection limit (2.5 fmol/μL), an ideal enrichment capacity (120 mg/g), and enrichment recovery (103.5%±6.6% and 101.5%±10.4%). We identified a total of 86 glycopeptides derived from 53 glycoproteins with 94 *N*-glycosylation sites from only 1 μL human serum. The O-T-D-COFs exhibited a good glycopeptide separation and enrichment potential, indicating that the COF material has promising application potential in glycoproteomics.

共价有机骨架材料(covalent organic frameworks,简称COFs)是有机单体通过共价键结合构成的一种新型的有序结晶聚合物^[1,2]^。作为一种具有周期性网络结构的有机多孔材料,COFs不仅具有多样的孔洞结构和大的比表面积,以及优异的隔音性、隔热性和渗透性,而且具有更加均一的孔洞尺寸与稳定的结构构型,可进行合理的设计和组装^[3,4,5]^。在其骨架上通过各种化学反应接枝上许多功能基团,可大大提升材料功能的多样性。目前,COFs材料吸引了众多研究者的兴趣,设计及合成了越来越多的COFs材料,研究主要集中在合成不同连接方式和不同单体组成的COFs材料,以及COFs材料功能化修饰等方面^[6,7]^。

继基因组学之后,蛋白质组学成为新的研究热点。作为生命活动的主要承担者,蛋白质的多种翻译后修饰(糖基化、磷酸化、甲基化、泛素化修饰等)参与或者调控生物体内重要的生理、病理过程,并在人体不同的健康状况或生长时期发生改变^[8,9,10]^。其中,蛋白质的糖基化修饰是上述几种修饰蛋白质中最常见、最重要的翻译后修饰之一^[11,12]^。糖蛋白组学研究有几个任务,分别为发生糖基化的蛋白质的鉴定、糖肽结构的解析、糖基化位点的鉴定和研究糖基化对蛋白质功能的影响^[13,14,15,16]^。生物质谱技术的发展使人们能在分子水平上进行糖蛋白或糖肽的有效监测^[17,18]^。但是,和其他蛋白质相比,糖蛋白的数量较低,酶解后的糖肽丰度就更低了,因此,需要一些手段对糖肽进行有效的富集,以提高其检测丰度^[19,20,21,22]^。开发新的富集方法和富集材料是糖蛋白组学领域的研究热点^[23,24,25,26,27,28,29,30]^。

COFs有序坚固的框架孔道结构,提供了作为糖肽富集材料载体的条件,同时基于载体骨架结构的功能化修饰也将大大提升糖肽富集分离效率^[31,32,33,34]^。Zhang课题组^[35]^将一种新型COF功能化的磁性石墨烯复合物(MagG@COF-5)作为超敏感基质用于识别糖肽,通过LC-MS/MS分析,成功地检测到了232个独特的*N*-连接糖肽和85个特征糖蛋白。*N*-连接糖肽已被证明是治疗各种疾病的关键生物标志物,Zhang课题组^[36]^研究出的还原型谷胱甘肽功能化银纳米粒子修饰COFs材料应用于*N*-连接糖肽的富集研究中,提高了COFs的富集选择性,并提供了一种新的方法。Wu课题组^[37]^使用磁性胶体纳米晶体簇(MCNC)作为核心,利用后修饰策略制备了另一种谷胱甘肽功能化的高亲水性磁性COFs,用于人唾液中内源性*N*-连接糖肽的富集,同样表现出优异的糖肽富集性能,如高灵敏度及选择性、良好的尺寸排阻效应和可重复使用性等。但目前将结构修饰功能化的COFs材料应用于糖蛋白组学的研究还在起步阶段,相关研究较少,也存在糖肽选择性不足、相互特异性作用不强等问题。

本文以开发新的糖肽富集材料为目的,结合COFs材料的功能化修饰,以2,5-二甲氧基苯-1,4-二甲醛(DMTA)和1,3,5-三(4-氨苯基)苯(TAPB)为单体,通过溶剂热法,首先制备得T-D-COF,再用亚氯酸钠溶液进行氧化,发展出一种新的材料O-T-D-COF。将该材料进行糖肽的富集应用,在复杂体系验证富集选择性,以及考察富集检测限、富集容量和回收率。基于COFs功能化修饰的新材料,有望为开发糖肽富集新材料提供新的思路,为糖蛋白组学提供了一种选择性更优、可控性更好的糖肽富集新思路。

## 1 实验部分

### 1.1 仪器、试剂与材料

TAPB、DMTA、正丁醇、邻二氯苯、冰醋酸、碳酸氢铵、尿素购自麦克林生化科技有限公司(中国上海)。甲醇购自国药集团化学试剂有限公司(中国上海)。四氢呋喃、硼氢化钠、乙腈(ACN)、三氟乙酸(TFA)购自上海阿拉丁生化科技股份有限公司(中国上海)。人血清免疫球蛋白G(IgG)、牛血清白蛋白(BSA)、二硫苏糖醇(DTT)、碘代乙酰胺(IAA)、胰蛋白酶、*N*-糖苷酶F(包含Glycobuffer)、2,5-二羟基苯甲酸(DHB)购自Sigma-Aldrich(美国)。此外,实验用水为Milli-Q超纯水系统(美国Millipore公司)制备的超纯水。人血清样本采集自健康人,按照标准临床程序从上海交通大学附属第六人民医院获得。

扫描电子显微镜(SEM)的型号为S-3400N (Hitachi,日本),透射电子显微镜(TEM)的型号为JEOL JEM-1400(JEOL,日本),固体核磁测试采用500MHz核磁共振波谱仪(Bruker,瑞士),红外光谱采用全反射傅立叶红外光谱仪(ATR-FTIR)型号为Nicolet 6700 (Thermo Scientific,美国)。

### 1.2 COFs的制备

称取TAPB 0.526 g(1.5 mmol),装于50 mL三口烧瓶中;量取10 mL邻二氯苯和10 mL正丁醇,混合均匀后加入到三口烧瓶中,将烧瓶放在搅拌器上充分搅拌。接着,称取0.436 g(2.25 mmol)DMTA,倒入三口烧瓶中,用移液枪吸取2 mL(6 mol)冰醋酸,装入三口烧瓶中作为催化剂。用橡皮塞把三口烧瓶的两端塞紧,中间放上冷凝管,冷凝管的上方接上油封装置,将针头插入橡皮塞后插到液面以下,通入氮气作为保护,调节氮气流量为每秒一个气泡。将三口烧瓶放入油浴锅中,120 ℃冷凝回流,磁力搅拌。72 h后停止搅拌和加热,待装置冷却到室温后,抽滤得到黄色的固体产物。产物用四氢呋喃重复洗涤7次,去除杂质。洗涤后的产物放入30 ℃干燥箱隔夜烘干,得到黄色粉末,标记为T-D-COF。称取47.08 mg T-D-COF粉末,溶于4 mL二恶烷中制成悬浮液。向悬浮液中依次加入2.548 mL(24.0 mmol)2-甲基-2-丁烯、400 μL浓度为3.3 mol/L的亚氯酸钠水溶液、137.6 μL(2.4 mmol)冰醋酸。所用的T-D-COF、2-甲基-2-丁烯、亚氯酸钠和冰醋酸的物质的量之比为1∶100∶5.5∶10。在室温避光的条件下,将两相悬浮液持续搅拌20 h,所得产物为紫黑色固体。反应后的体系通过过滤分离,得到的固体依次用40 mL超纯水、40 mL 10%(体积分数)硫代硫酸钠、40 mL超纯水和40 mL丙酮洗涤,产物置于60 ℃真空干燥箱中干燥10 h,得到紫色粉末,标记为O-T-D-COF,制备过程如图1所示。

**图 1:**
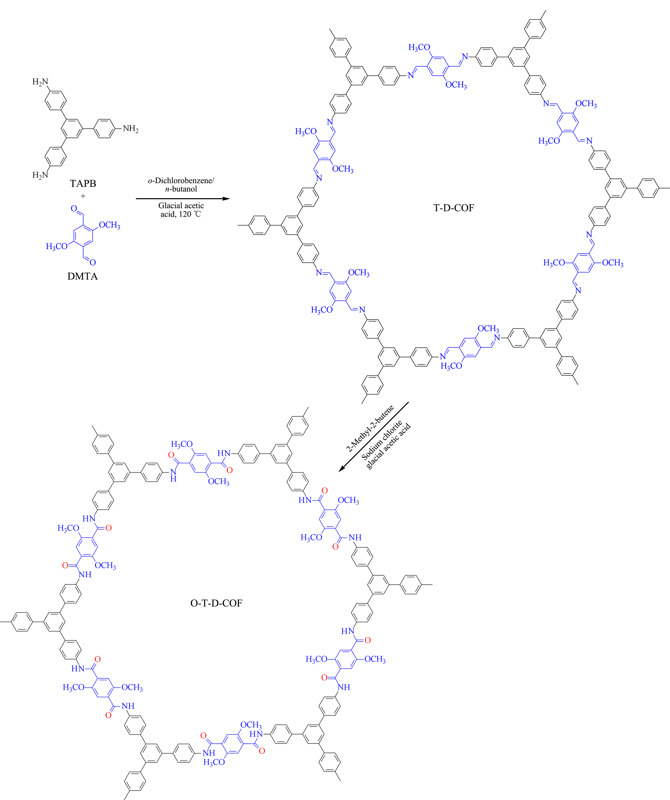
O-T-D-COF的合成路线

### 1.3 蛋白酶解

称取1 mg IgG (或1 mg BSA),用100 μL含有8 mol尿素的50 mmol碳酸氢铵的溶液将IgG完全溶解,向溶液中加入5 μL DTT(200 mmol),在恒温56 ℃的条件下振荡45 min。取20 μL IAA(200 mmol)避光条件下配制,加入到振荡后的溶液中,避光条件下静置30 min。然后向溶液中继续加入碳酸氢铵溶液,直至溶液体积达到1 mL。向溶液中加入20 μg胰蛋白酶后,将溶液放置在37 ℃的培养器中,持续酶解17 h后向体系中加入5 μL甲酸(FA)使酶解反应停止。

### 1.4 糖肽的富集

使用离心法进行O-T-D-COF材料对糖肽的富集实验,在离心管中加入500 μg O-T-D-CO材料,加入100 μL上样液(ACN∶H_2_O∶TFA=90∶5∶5, v/v/v),超声1 min、振荡5 min进行润洗,离心去除上清液;取IgG样品(之前已分装旋干)6 μL,用180 μL上样液重溶;20 μL上样液溶解O-T-D-COF材料,将溶解了O-T-D-COF材料的上样液转移至IgG的溶液中,超声1 min、振荡30 min进行孵化,离心去除上清液;将上样后的材料分散在200 μL的淋洗液(ACN∶H_2_O∶TFA=90∶9.9∶0.1, v/v/v)中,超声1 min、振荡5 min进行淋洗,离心去除上清液,重复该步骤3次;取10 μL洗脱液(H_2_O∶TFA=99∶1, v/v)加入离心管中,超声1 min、振荡20 min将富集在材料中的糖肽分离出来,离心后保留上清液。复杂体系中的富集、富集检测限、富集容量和回收率操作参照以上操作流程。

### 1.5 脱糖基实验

人血清蛋白酶解液的富集按照1.4节进行操作,将糖肽样品冻干后加入17 μL水和2 μL Glycobuffer,混匀,再加入1 μL *N*-糖苷酶F(PNGase F)。37 ℃下振荡17 h后,离心浓缩,重溶于10 μL含0.1%(v/v)的FA溶液中,等待进一步进行分析。

### 1.6 分析条件

糖肽样品洗脱液的检测使用基质辅助激光解吸电离-飞行时间-质谱(简称MALDI-TOF/TOF-MS, AB Sciex,美国),在反射正离子模式下进行分析。取1 μL富集后的洗脱液,点在靶上,待溶液自然干燥后,取1 μL质量浓度为25 mg/mL的DHB基质溶液(ACN∶H_2_O∶H_3_PO_4_=70∶29∶1, v/v/v)覆盖在样品点上。质谱仪的检测范围是*m/z* 2000~3500,激光强度是3800 μJ。得到的结果利用软件Data Explorer (TM) Software进行数据分析,获得质谱图峰的信息,其中峰的分辨率设置为0.95, *S/N*>3。

人血清提取蛋白酶解液的糖基化肽段/蛋白质采用赛默飞高分辨液相色谱-质谱联用仪Thermo EASY-nanoLC 1000 Nano HPLC System (Thermo Fisher Scientific,德国)分析,该仪器配备了纳升液相色谱和Q-Exactive plus质谱仪。采用同公司纳米反相C18柱(15 cm×75 μm)。流动相A为ACN-FA(99.9∶0.1, v/v), B为H_2_O-FA (99.9∶0.1, v/v)。洗脱方式如下:0%B~4%B, 2 min; 4%B~35% B, 90 min; 35%B~45% B, 10 min; 45%B~90%B, 5 min; 90% B, 5 min;最后用100%A冲洗柱子15 min。流动相流速为300 nL/min,柱温为25 ℃,进样量为1 μL。Q-Exactive plus质谱的实验参数为:电喷雾电压为2.0 kV,扫描范围为*m/z* 400~2000; MS/MS采集在Orbitrap中进行,分辨率为35000(*m/z* 200)。数据处理:采用搜索软件Protein Discovery 1.4对数据进行搜库,搜索引擎为Sequest HT,该搜索引擎可搜索所有数据库包括人的UniProtKB/SwissProt数据库。

## 2 结果与讨论

### 2.1 O-T-D-COF材料的合成

本实验用到的合成方法是溶剂热法。本实验合成的O-T-D-COF材料是一种具有二维(2D)结构的材料,反应单体分别是TAPB和DMTA。这两种单体中分别含有醛基和氨基,通过共聚缩合反应生成的席夫碱构成了T-D-COF材料的框架,每6份单体组合成一个六边形的大环,这些大环再通过层层堆叠形成2D的管状结构。该反应对原料和产物的溶解性要求较高。在溶剂中,原料慢慢溶解到溶剂中参与反应,这样有利于合成的材料具有有序的结构,并且能够最大限度地减少材料结构上的缺陷。因此本实验选择的溶剂是正丁醇和邻二氯苯,这二种溶剂对上述两种单体的溶解性都比较小,有利于原料缓慢溶解到溶剂中。冰醋酸作为反应的催化剂可以活化反应物中的羰基,有利于脱水缩合反应。然后对合成后的COFs材料进行氧化处理,可以提高材料的富集性能。2D层状的O-T-D-COF材料的结构稳定性来自于层与层的作用力,夹层间的作用力是形成堆积结构的重要作用力,直接影响到材料的孔隙率和结晶性。在材料中引入的甲氧基就很好地加强了层与层之间的作用力,甲氧基处于苯基的边缘上,每个氧原子上的二对孤电子对转移到苯环中心,这就使得层与层之间电荷排斥力减小,从而增强了夹层间的作用力,改善了O-T-D-COF材料的稳定性和结晶性。

### 2.2 O-T-D-COF材料的表征

通过扫描电镜观察O-T-D-COF材料的形貌,图2a和2b是材料O-T-D-COF在扫描电镜下成像的图片,从图中可以看到O-T-D-COF材料呈略规则状聚集在一起。放大倍数为5 k倍时,从图2a可以看出O-T-D-COF材料的空间分布很密集,单个单元尺寸较小。放大倍数为18 k倍时(见图2b)可以看到每个单元之间结合比较紧密,结构较为规则。图2c是O-T-D-COF材料的固体核磁表征图,从图中的1号峰位置可以看出材料被部分氧化。红外光谱进一步表征T-D-COF与O-T-D-COF材料的特征峰差异(见图2d)。在材料氧化后,谱图上1668 cm^-1^附近的峰是酰胺键的C=O伸缩振动,1515 cm^-1^出现新的伸缩振动峰,该峰为酰胺基带的峰,这一结果也说明了COFs材料已被成功制备。

**图 2:**
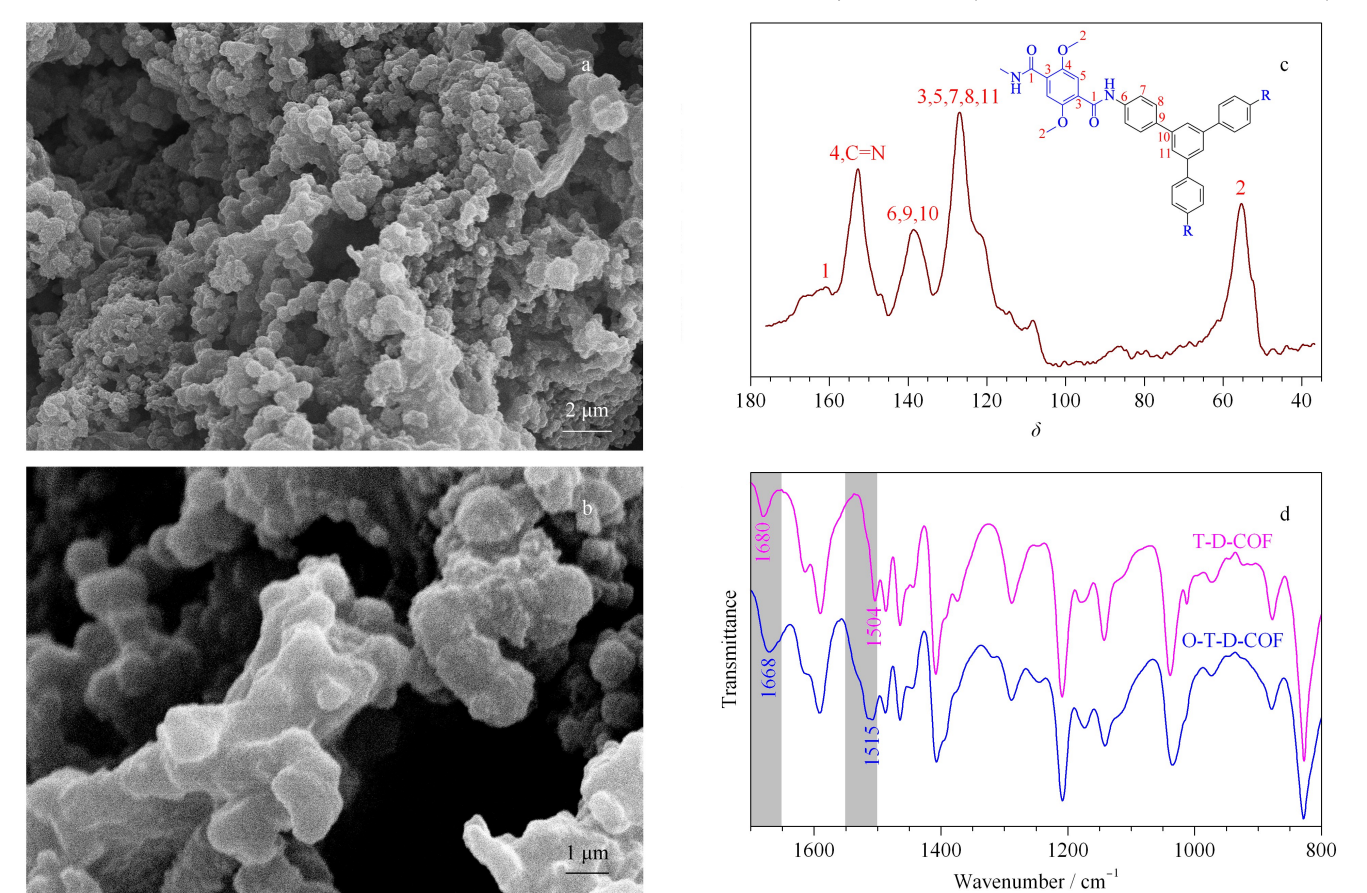
O-T-D-COF材料放大(a)5000倍和(b)18000倍的SEM图及(c)固体核磁^13^C谱和(d)红外光谱图

通过透射电镜观测物质微观形貌时,能够看到其聚集状态,分辨率高时还能看到晶体的晶格条纹,从而可以测量材料的孔径大小。图3为O-T-D-COF材料在透射电镜中形成的图像,从图中可以看到,材料中单元的规则结构呈现密集状态,在高倍分辨率下能看到晶体的晶格条纹明暗对比,单元呈中空状态,表明该材料具有规则的孔道结构。

**图 3:**
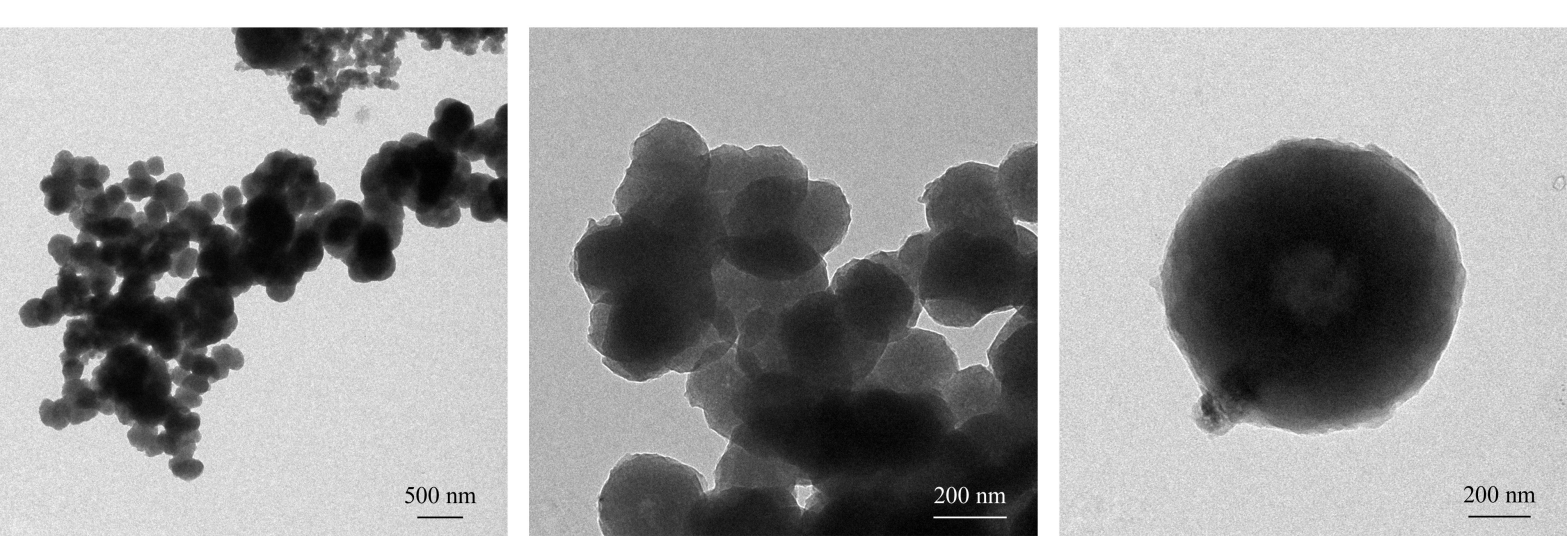
O-T-D-COF材料的TEM图

### 2.3 O-T-D-COF材料的糖肽富集条件优化

IgG是人体免疫系统中的一种保护物质,对IgG的研究在疾病诊断等领域有重要意义。IgG主要分为IgG 1和IgG 2两个亚型,都具有糖基化的位点,且位点上均连接了一个以五碳作为核心的聚糖。本文利用MALDI技术测定富集洗脱液中的糖肽,通过谱图分析探讨富集效果。

在上样液酸度条件的优化中,使用了4种不同酸度的上样液进行实验。其中TFA的体积分数分别是:0.1%、1%、3%、5%,配制成乙腈体积分数为90%的酸性溶液。如图4a所示,当上样液中TFA的体积分数为0.1%、1%和3%时,6条糖肽的信号峰都较弱;相比之下,在TFA体积分数在5%时,每一个柱形都明显比前3组对应柱形的长度更长,说明此酸浓度下的上样条件更有利于富集,因此得出上样液中TFA的最佳体积分数为5%。

**图 4:**
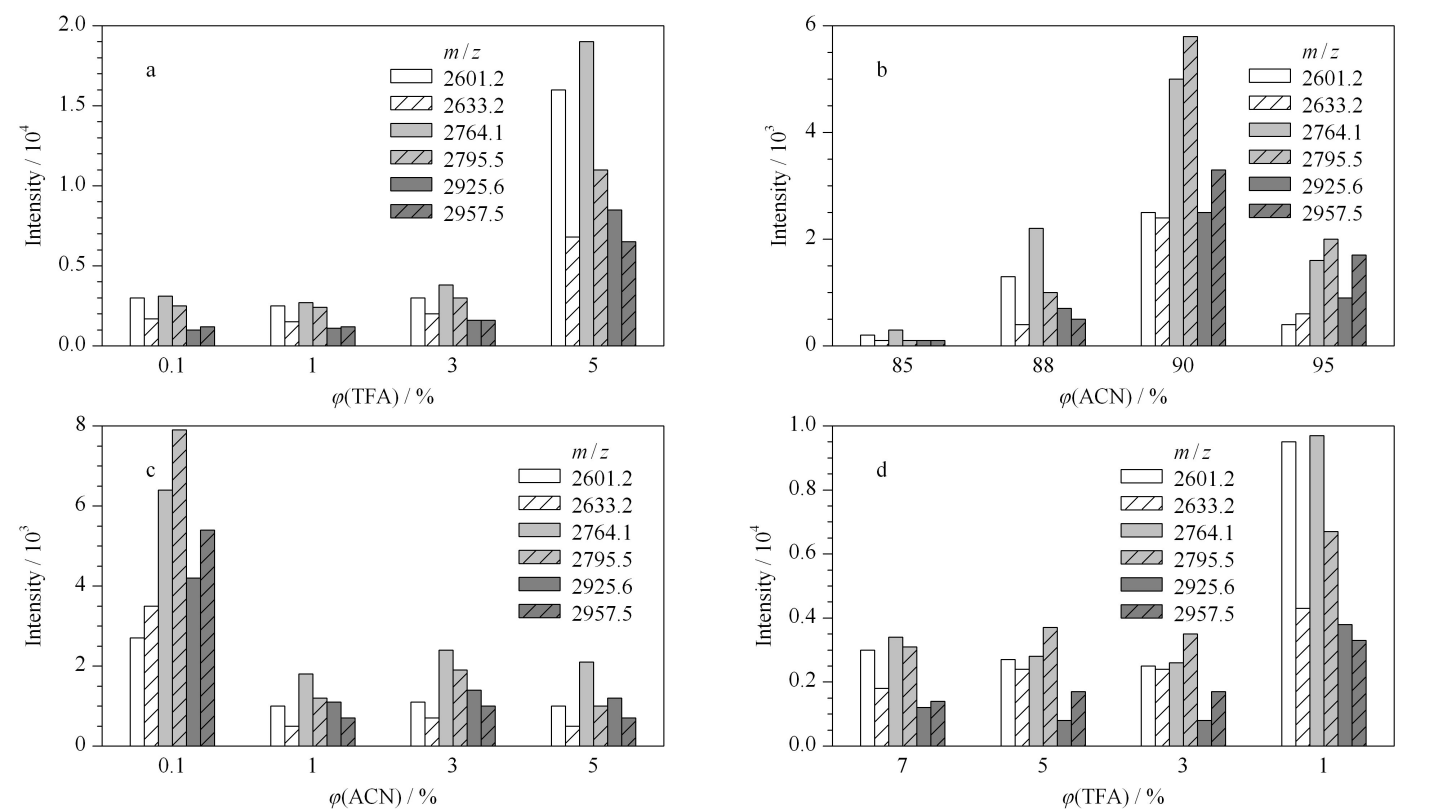
(a)上样液的酸度、(b)上样液中的乙腈体积分数、(c)淋洗液的酸度、(d)洗脱液的酸度对富集效果的影响

考察上样液中的乙腈的体积分数,分别设为85%、88%、90%、95%, TFA的体积分数固定为5%。如图4b所示,随着上样液中乙腈浓度的增加,信号强度先增加后减少。当乙腈达到90%时,6条肽的信号均比其他3组的结果好,说明此时富集效果最好。这一结果可以说明,乙腈低于90%时,糖肽不能很好地与O-T-D-COF材料结合,而乙腈高于90%时,糖肽在上样液中的溶解性较差,从而降低了富集效果。因此得出结论,上样液中最佳的乙腈体积分数为90%。

为了更好地淋洗与分离材料上的非糖肽物质和其他杂质,进一步优化了淋洗液。考察不同酸度的淋洗液对富集效果的影响,分别配制了TFA体积分数为0.1%、1%、3%和5%的淋洗液,淋洗液中ACN的体积分数固定为90%。从图4c可以看出,淋洗液中TFA的体积分数为0.1%时,糖肽的信号最好,随着淋洗液酸度的增加,糖肽信号强度逐渐降低。这一结果说明,淋洗液的酸度变高可能会造成糖肽的流失,不利于材料对糖肽的富集。

最后,为了考察洗脱液中酸浓度对富集效果的影响,分别配制了4种洗脱液条件,其中TFA的体积分数分别为7%、5%、3%、1%。从图4d可以看出,只有第4组的6条信号峰信号较强,此时洗脱液中酸的体系分数为1%。因此得出结论,洗脱液中TFA的最佳体积分数为1%。

以上的优化实验表明,O-T-D-COF材料对糖肽的保留是结合亲水作用和静电作用的结果。O-T-D-COF对糖肽有较强的亲水作用,对糖肽有很强的保留。通过亲水模式的洗脱富集,以及淋洗操作条件优化,可以很好地去除非糖肽。图5是未经过材料富集的糖肽酶解液的质谱图和经材料在最优条件下富集的IgG糖肽样品的质谱图的对比。图5a中,由于糖肽酶解液中存在高丰度的非糖肽及杂质信号,这些物质在质谱检测中会严重干扰和抑制糖肽的信号,使得质谱仪不容易检测到糖肽的信号,未经材料富集的糖肽的信号峰只有4条,丰度低且选择性差。而图5b中,一共可以鉴定到32条糖肽的信号峰,并且几乎没有非糖肽信号的干扰,很好地提高了糖肽富集选择性与检测丰度。

**图 5:**
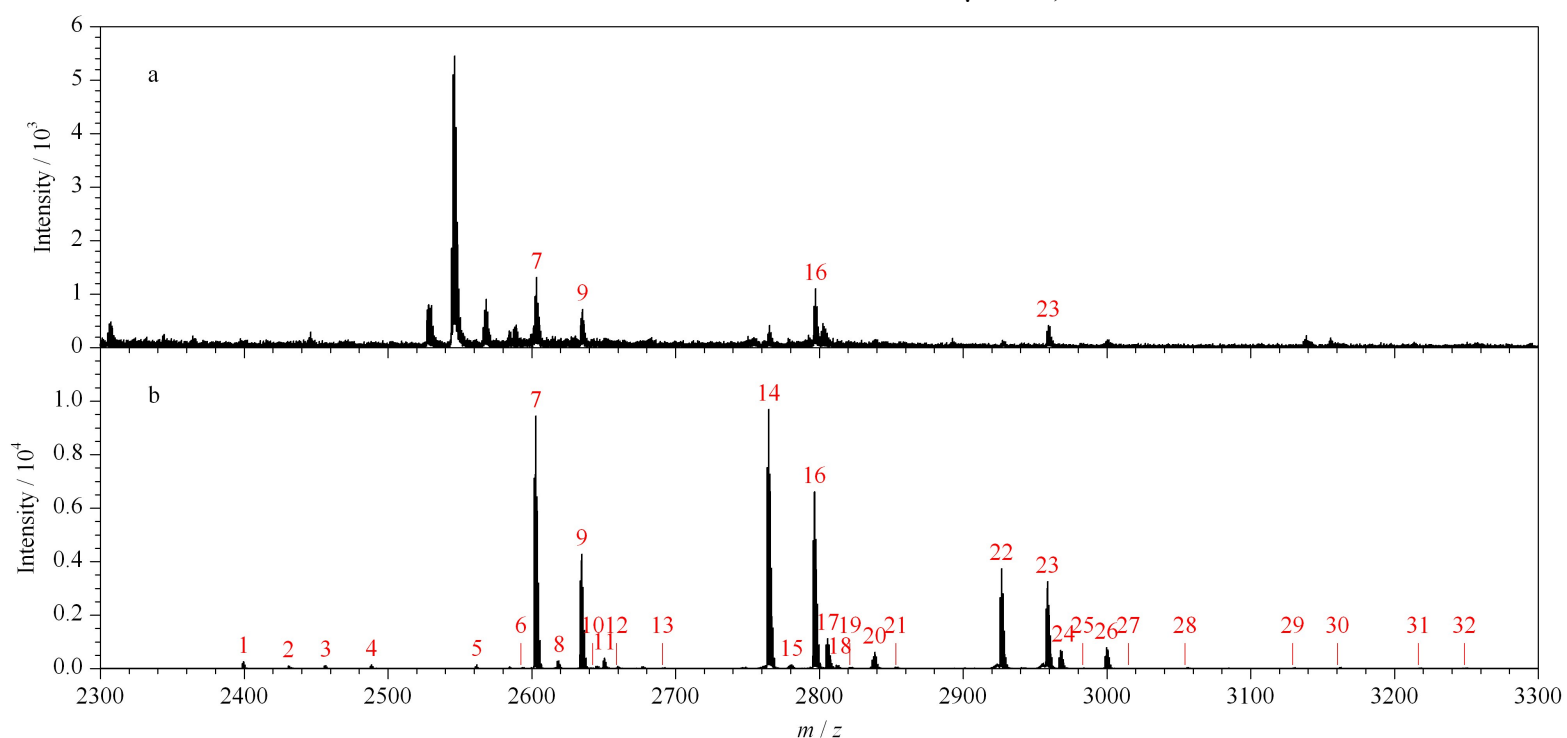
富集(a)前和(b)最优条件下富集的糖肽质谱图

### 2.4 O-T-D-COF材料的富集选择性验证

为考察O-T-D-COF材料在复杂体系中对富集糖肽的选择性,将IgG和BSA的酶解液混合,以此为研究对象,考察O-T-D-COF材料在复杂体系中的糖肽富集情况。图6a为IgG和BSA酶解液物质的量之比为1∶10的糖肽富集结果,可以鉴定到30条糖肽的信号峰,并且信号丰度佳。图6b为IgG和BSA酶解液物质的量之比为1∶50的糖肽富集结果,可以鉴定到24条糖肽信号峰,依然保持了较好的富集丰度及富集选择性。以上结果都展示出O-T-D-COF材料在糖肽富集领域较好的应用潜力。

**图 6:**
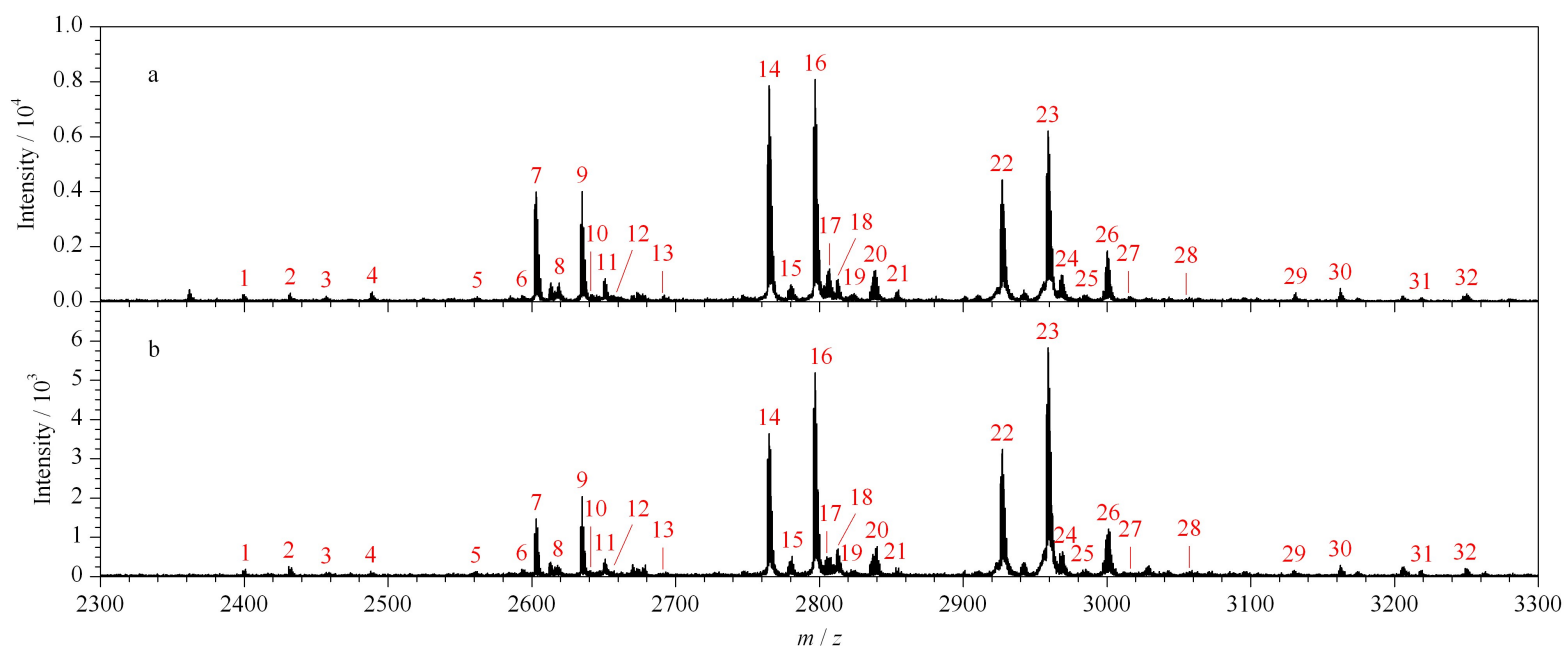
复杂体系的糖肽富集质谱图

### 2.5 O-T-D-COF材料的富集检测限

在糖肽富集实验中的检测限是指在满足实验要求的情况下,待测糖肽样品的最小浓度。O-T-D-COF材料检测限的大小代表了材料在低丰度糖肽样品中,对糖肽的特异性吸附能力大小。因此,在富集检测限的研究中,分别配制了5 fmol/μL和2.5 fmol/μL的低浓度IgG酶解液溶液进行富集操作。图7a为5 fmol/μL样品的富集结果,可以鉴定到16条糖肽的信号峰,其中6条高丰度糖肽的信号较强。图7b图为2.5 fmol/μL样品的富集结果,仍能鉴定到12条糖肽信号峰,且6条高丰度糖肽的信号较强。从这一结果可以得出,当样品的浓度低至2.5 fmol/μL时,依然可以进行糖肽信号的有效鉴定,说明O-T-D-COF材料在低浓度的糖肽样品中仍然对糖肽有较好的特异性识别能力。

**图 7:**
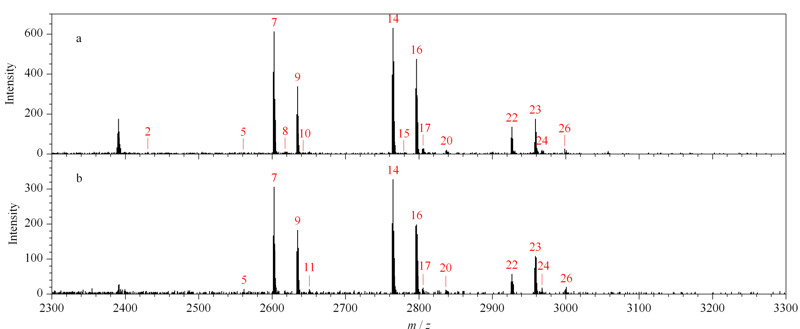
(a)5 fmol/μL和(b)2.5 fmol/μL的IgG酶解液的富集质谱图

### 2.6 O-T-D-COF材料的富集容量

O-T-D-COF材料的富集容量是一项重要的考察指标,实验中固定糖肽样品的质量为6 μg,以不同质量(10、20、30、40、50、100、150和200 μg)的材料进行富集。图8为不同材料用量的实验结果,纵坐标为质谱信号的强弱。从图8中可以看出,随着材料量的增加,质谱信号逐渐增加;当材料质量增至50 μg时6条信号峰的强度最强;此后,信号强度趋于平缓。因此,得出O-T-D-COF材料对糖肽的富集容量为120 mg/g。

**图 8:**
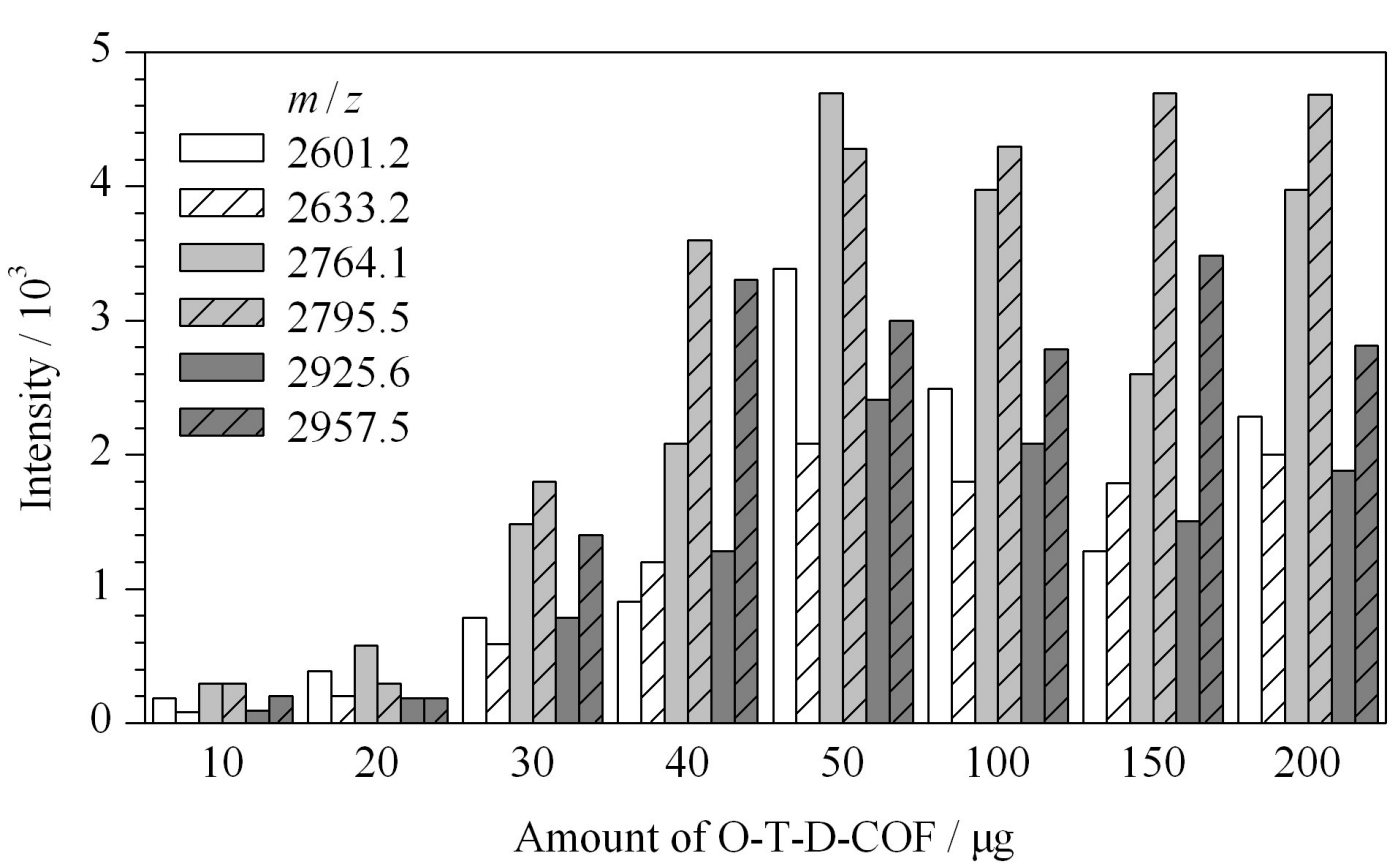
不同质量O-T-D-COF材料富集6 μg IgG酶解液的结果

### 2.7 O-T-D-COF材料的富集回收率

通过稳定同位素二甲基标记的方法进行了O-T-D-COF材料富集回收率的考察。该实验需要通过两步进行,第一次富集的样品是3 μg氘代二甲基标记的IgG酶解液,将第一次实验所得洗脱液冻干,与3 μg二甲基标记的IgG酶解液混合得到第二次待富集样品。经第二次富集所得的洗脱液经过冻干,脱糖基后用于MALDI分析,本实验平行做3组进行对比。IgG的两条糖肽序列分别为EEQYN#STYR和EEQFN#STFR(N#为修饰位点)。经过脱糖基后的两条糖肽的*m/z*分别是1158.5和1190.5,由二甲基标记过的两条糖肽的*m/z*分别是1186.6和1218.5(标记为轻样);用氘代二甲基标记后两条脱糖基肽段的*m/z*分别增加至1190.6和1222.5(标记为重样)。如图9所示,轻样和重样的信号峰同时出现在谱图中,同一条糖肽的轻样和重样信号峰的信号强度之比即为O-T-D-COF材料的回收率。

**图 9:**
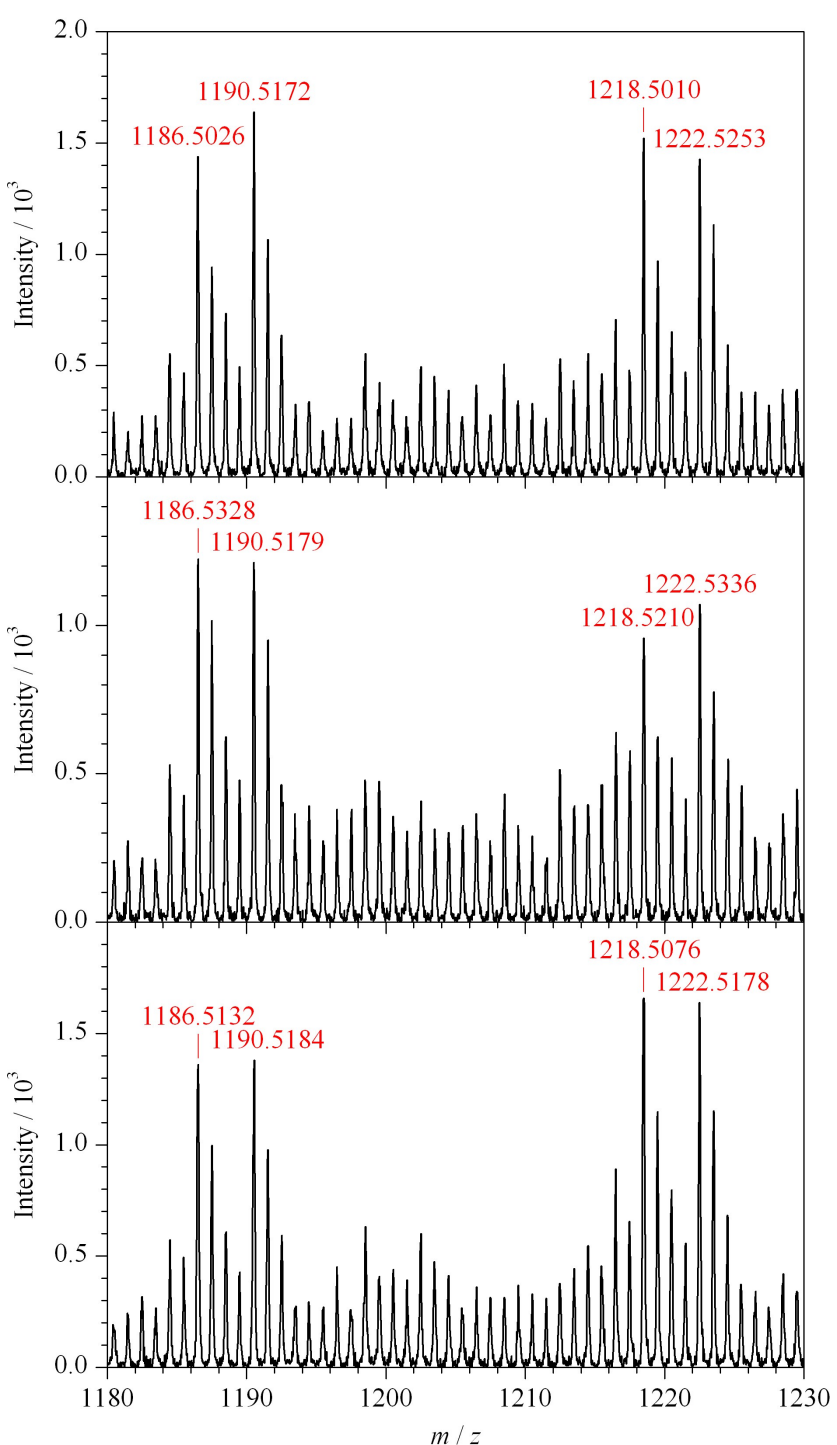
从IgG酶解液回收轻/重二甲基标记的去糖基化肽的3组平行试验

将3组平行实验的结果统计到表1中,再计算其平均值分别为103.5%和101.5%,该值即为O-T-D-COF材料的回收率。

**表 1:** 人IgG酶解液经O-T-D-COF材料富集后两个去糖基化肽的回收率(*n*=3)

No.	Recoveries/%	
	EEQFN#STFR (m/z 1158.5)	EEQYN#STYR (m/z 1190.5)
1	110.1	93.8
2	99.0	111.9
3	101.4	98.8
Average	103.5±6.6	101.5±10.4

### 2.8 O-T-D-COF材料在实际样品中的应用

从前期的结果可知,该材料拥有良好的富集性能,因此被进一步应用于人血清样品的糖肽富集。人血清样品是目前糖肽富集中研究最多的生物样品,包含了丰富的生理和病理状况信息。但血清中,糖蛋白的分析因其丰度低具有较大的难度,需要用有效的样品富集前处理操作,才能将其检测到。我们利用高分辨液相色谱-质谱联用仪对O-T-D-COF材料富集结果进行了检测与分析,得到了血清中来自53个*N*-糖蛋白中的86个*N*-糖肽序列,并鉴定到了94个*N*-糖基化位点(见附表1,详见http://www.chrom-China.com/)。

## 3 结论

本文利用溶剂热合成法制备了一种2D层状、具有孔洞结构的O-T-D-COF材料,结合SEM和TEM对其进行了表征。将制备的材料进行了氧化后处理以提高材料对糖肽富集的效率,且成功应用于糖肽的选择性富集。建立了一个较为完整的体系,探讨了富集过程中上样液条件、淋洗液条件、洗脱液条件等对富集结果的影响,对富集条件进行了优化。并在复杂样品体系进行了富集选择性的验证,表明O-T-D-COF材料在IgG和BSA酶解液的物质的量之比为1∶50时,具有良好的糖肽富集选择性。同时。具有较低的检测限(2.5 fmol/μL)、较高的富集容量(120 mg/g),及较好的富集回收率(103.5%、101.5%)。最后,我们将O-T-D-COF应用于实际样品中的糖肽富集,得到了血清中来自53个*N*-糖蛋白中的86个*N*-糖肽序列,并鉴定到了94个*N*-糖基化位点。这些都展现了O-T-D-COF材料在糖肽富集领域的应用潜力。

## References

[1] Feng X, Ding X S, Jiang D L. Chem Soc Rev, 2012,41(18):6010 22821129 10.1039/c2cs35157a

[2] Geng K Y, He T, Liu R, et al. Chem Rev, 2020,120(16):8814 31967791 10.1021/acs.chemrev.9b00550

[3] Ding S Y, Wang W. Chem Soc Rev, 2013,42(2):548 23060270 10.1039/c2cs35072f

[4] Huang N, Wang P, Jiang D. Nat Rev Mater, 2016,1(10):16068

[5] Liang R R, Jiang S Y, Zhao X. Chem Soc Rev, 2020,49(12):3920 32427238 10.1039/d0cs00049c

[6] Wang X, Ye N S. Electrophoresis, 2017,38:3059 28869768 10.1002/elps.201700248

[7] Chen X, Geng K, Liu R, et al. Angew Chem Int Edit, 2020,59(13):5050 10.1002/anie.201904291 31144373

[8] Mann M, Jensen O N. Nat Biotechnol, 2003,21(3):255 12610572 10.1038/nbt0303-255

[9] Huang J F, Wang F J, Ye M L, et al. J Chromatogr A, 2014,1372:1

[10] Zhang Y, Zhang C, Jiang H C, et al. Chem Soc Rev, 2015,44(22):8260 26258179 10.1039/c4cs00529e

[11] Dube D H, Bertozzi C R. Nat Rev Drug Discov, 2005,4(6):477 15931257 10.1038/nrd1751

[12] Hart G W, Copeland R J. Cell, 2010,143:672 21111227 10.1016/j.cell.2010.11.008 PMC3008369

[13] Moremen K W, Tiemeyer M, Nairn A V. Nat Rev Mol Cell Bio, 2012,13(7):448 22722607 10.1038/nrm3383 PMC3934011

[14] Ongay S, Boichenko A, Govorukhina N, et al. J Sep Sci, 2012,35(18):2341 22997027 10.1002/jssc.201200434

[15] Gaunitz S, Nagy G, Pohl N L B, et al. Anal Chem, 2017,89(1):389 28105826 10.1021/acs.analchem.6b04343 PMC5609817

[16] Qing G Y, Yan J Y, He X N, et al. Trend Anal Chem, 2020,124:115570

[17] Pan S, Chen R, Aebersold R, et al. Mol Cell Proteomics, 2011, 10(1): R110.003251 10.1074/mcp.R110.003251 PMC3013464 20736408

[18] Liu S, Jiang X T, Shang Z, et al. Anal Chim Acta, 2020,1123:18 32507236 10.1016/j.aca.2020.04.063

[19] Witze E S, Old W M, Resing K A. Nat Meth, 2007,4(10):798 10.1038/nmeth1100 17901869

[20] Cummings R D, Pierce J M. Chem Biol, 2014,21(1):1 24439204 10.1016/j.chembiol.2013.12.010 PMC3955176

[21] Palaniappan K K, Bertozzi C R. Chem Rev, 2016,116(23):14277 27960262 10.1021/acs.chemrev.6b00023 PMC5327817

[22] Huang B Y, Yang C K, Liu C P, et al. Electrophoresis, 2014,35(15):2091 24729282 10.1002/elps.201400034

[23] Qing G Y, Lu Q, Xiong Y T, et al. Adv Mater, 2017,29(20):1604670 10.1002/adma.201604670 28112833

[24] Cai T P, Rahman A F M M, et al. Microchim Acta, 2017,184(8):2629

[25] Chen Y X, Sheng Q Y, Hong Y Y, et al. Anal Chem, 2019,91(6):4047 30794378 10.1021/acs.analchem.8b05578

[26] Chen C, Kang H J, Zhang X F, et al. Chinese Journal of Chromatography, 2019,37(8):845 10.3724/SP.J.1123.2019.03028 31642255

[27] Zheng H J, Jia J X, Li Z, et al. Anal Chem, 2020,92(3):2680 31977188 10.1021/acs.analchem.9b04691

[28] Lu Q, Chen C, Xiong Y T, et al. Anal Chem, 2020,92(9):6269 32233396 10.1021/acs.analchem.9b02643

[29] Zhang H Q, Lv Y Y, Du J, et al. Anal Chim Acta, 2020,1098:181 31948582 10.1016/j.aca.2019.11.012

[30] Zheng X T, Wang X, Zhang F S, et al. Chinese Journal of Chromatography, 2021,39(1):15 10.3724/SP.J.1123.2020.05036 PMC9274847 34227355

[31] Ma Y F, Yuan F, Zhang X H, et al. Analyst, 2017,142(17):3212 28765847 10.1039/c7an01027c

[32] Wang H P, Jiao F L, Gao F Y. J Mater Chem B, 2017,5(22):4052 32264138 10.1039/c7tb00700k

[33] Gao C H, Lin G, Lei Z X. J Mater Chem B, 2017,5(36):7496 32264225 10.1039/c7tb01807j

[34] Ding F J, Chu Z Y, Zhang Q Q, et al. Anal Chim Acta, 2019,1057:145 30832913 10.1016/j.aca.2018.12.063

[35] Wang J X, Li J, Gao M X, et al. Nanoscale, 2017,9(30):10750 28715013 10.1039/c7nr02932b

[36] Ma Y F, Wang L J, Zhou Y L, et al. Nanoscale, 2019,11(12):5526 30860530 10.1039/c9nr00392d

[37] Luo B, He J, Li Z Y, et al. ACS Appl Mater Interfaces, 2019,11(50):47218 31750645 10.1021/acsami.9b15905

